# Antagonistic potential and analytical profiling of plant probiotic bacteria using chromatography and mass spectrometry techniques against *Botrytis cinerea* and *Fusarium oxysporum*

**DOI:** 10.1186/s40643-025-00853-0

**Published:** 2025-06-21

**Authors:** Gottumukkala Hiranmayee, Sarada Prasanna Mallick, Golamari Siva Reddy

**Affiliations:** 1 Department of Biotechnology, Koneru Lakshmaiah Education Foundation, Vaddeswaram, Guntur, Andhra Pradesh 522 502 India; 2 Department of Biotechnology, National Institute of Technology, Tadepalligudem, Andhra Pradesh 534101 India

**Keywords:** Antagonistic activity, Chemical characterization, Fourier transform infrared spectrum, Gas chromatography‒mass spectrometry, High-performance liquid chromatography, Phenols, Plant probiotics, Organic acids

## Abstract

Plant probiotics are bacteria that play a significant role in enhancing plant growth and health. To understand the interactions between plant probiotics and host plants, a comprehensive approach of antagonistic activity and analytical methods such as high-performance liquid chromatography (HPLC), gas chromatography‒mass spectrometry (GC‒MS), and Fourier transform infrared (FT‒IR) spectroscopy, were employed. The previously isolated bacterial strains, namely, *Corynebacterium accolens* strain CNTC Th1/57*, Bacillus rugosus* strain SPB7, *Lactobacillus pasteurii* DSM 23907 *and Cytobacillus firmus* strain NBRC 15306, were exposed to antagonistic testing against *Botrytis cinerea* and *Fusarium oxysporum.* Considering the results of the antagonistic activity both in vitro and statistically, the bacterial strains *Bacillus rugosus* strain SPB7 and *Lactobacillus pasteurii* DSM 23907 presented greater zones of inhibition. Hence these bacteria were moved to obtain comprehensive insights into the chemical composition. HPLC and GC‒MS resulted in the identification of phenols and organic acids. These results were further confirmed by FT-IR, which revealed a peak at 3500 cm^−1^ for *Bacillus rugosus* strain SPB7, where O–H, aromatic C-H and aromatic C = C stretching vibrations were also observed at 3069 and 1549 cm^−1^. The peak at 1736 cm^−1^ corresponds to the carboxyl group (-COOH) as the functional group with respect to *Lactobacillus pasteurii* DSM 23907. Further confirmation was performed by observing the other absorption bands at 3451 cm^−1^ and 2958 cm^−1^, indicating the presence of hydroxyl group (O–H) and alkyl group (C-H) stretching vibrations, thus confirming their potential for the production of phenols and organic acids, respectively, by bacteria. This findings would make a way to explore plant diseases, tolerance against pathogens, and also study ecological role of these bacteria in plant communities.

**Graphical Abstract:**

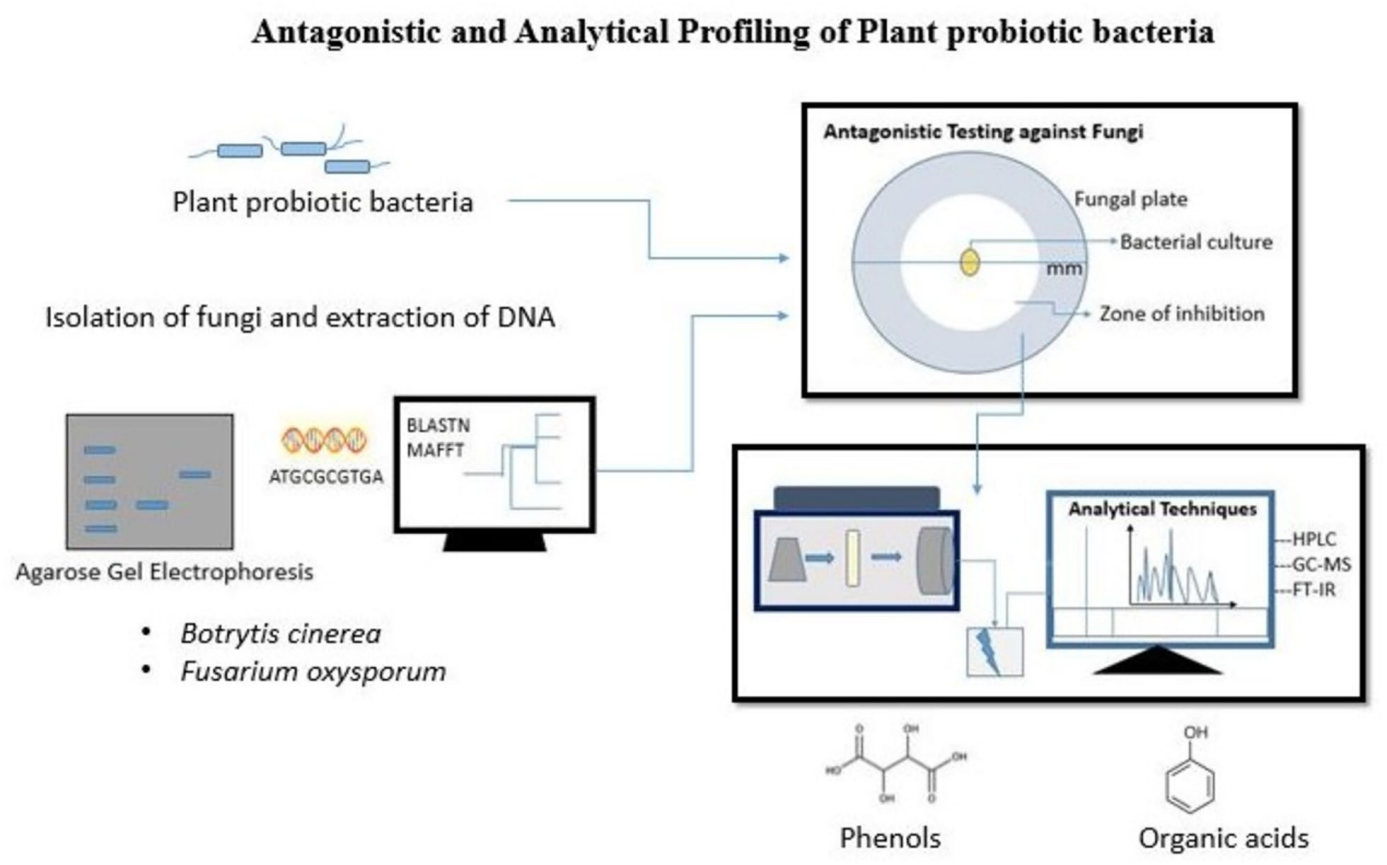

## Introduction:

Within soil, the rhizosphere stands out as the most dynamic zone, offering a versatile environment for nutrient cycling that promotes the proliferation of microorganisms (Solomon et al. 2024; Basu et al. 2021). Bacterial species residing in the rhizosphere exhibit a remarkable ability to shield plant roots from soil-borne pathogens and promote plant growth (Glick et al. 2007; Harish et al. 2009; Singh et al. 2011). Plant growth-promoting rhizobacteria (PGPR) can be viewed as proactive agents that mitigate environmental stressors and increase crop yield and quality even before harvest. Various bacteria, fungi, protozoa, and nematodes have exhibited antagonistic activity, indicating their potential for use in controlling root and foliar diseases as well as insect pests across multiple crop types (Mendes et al. 2011; Sehrawat and Sindhu 2019). Genera such as *Bacillus polymyxa, Bacillus pumilus, Bacillus cereus, and Bacillus oryzicola* are recognized for their ability to increase plant growth and vitality through diverse mechanisms, both direct and indirect (Hossain and Chung 2020; Idriss et al. 2022). Among plant growth-promoting rhizobacter (PGPR) strategies, *Bacillus* species stand out as promising candidates because of their wide-ranging antagonistic activity against phytopathogens, ability to form resilient spores, production of secondary metabolites, enzymatic breakdown of compounds, resilience to adverse conditions, and ability to promote plant growth. *Bacillus subtilis,* in particular, plays a pivotal role in enhancing plant growth and resilience against various biotic and abiotic stresses (Zehnder et al. 2001; Vessey 2003; Chandrasekaran and Chun 2016). Owing to their ability to benefit plants through enhanced productivity and immunity, these bacteria significantly affect plant growth. They achieve this by enhancing nutrient absorption, synthesizing biologically active phytohormones, and combating pathogens through the production of antibiotics, siderophores, and enzymes that breakdown fungal cell walls (Kuklinsky Sorbal et al. 2004; Frey-Klett et al. 2005; Hameeda et al. 2008; Singh et al. 2022; Orozco-Mosqueda et al. 2023; Khoso et al. 2004). On the other hand, the common rhizosphere microorganisms used in biocontrol include antagonistic fungi such as *Trichoderma viride* and *Trichoderma harzianum* as well as bacteria from genera such as *Pseudomonas fluorescens, Pseudomonas putida, Bacillus subtilis, Bacillus thuringensis, Streptomyces griseus, Streptomyces lydicus* (Ciancio et al. 2019). In addition to their antagonistic properties, these microbes enhance host health through various mechanisms, such as antibiosis. This process is considered the most potent mechanism for controlling plant diseases caused by microorganisms (Raajimakers and Mazzola 2012; He et al. 2021). Antibiosis occurs when antagonists emit chemical substances that either prevent or eliminate nearby prospective pathogens (Benitez et al. 2004; Irtwange 2006; Haggag and Mohamed 2007; Viterbo et al. 2007). Therefore, antagonism testing plays a vital role in assessing the effectiveness of plant probiotic bacteria. This confirms their ability to outcompete pathogens, thus preventing plant diseases through competitive exclusion. Furthermore, their biocontrol capacity can be evaluated, ensuring that they can directly counteract pathogens through mechanisms such as antibiosis or competition for attachment sites on plant surfaces (Davide et al. 2022). This test also considers compatibility with other beneficial microorganisms, ensuring a balanced soil microbiome. Antagonistic microbes in the rhizosphere protect host plants by directly suppressing the growth and proliferation of phytopathogens (Hariprasad et al. 2011; Prasanna Kumar et al. 2015). Some antagonists produce antibiotics to halt the spread of pathogens (Lee et al. 2006; Maheshwari 2010; Segarra et al. 2010; Brotman et al. 2012; Chauhan et al. 2016; Abd-Elhalim et al. 2023). For antagonists to serve as effective biocontrol agents, extracellular hydrolytic enzymes such as chitosanases, chitinases, cellulases, and/or proteases must breakdown the cell walls of pathogenic fungi (Spadaro and Droby 2016). This breakdown leads to cellular deformities, cytological damage, mycelial lysis, deformation, increased cell membrane permeability, and leakage of cytoplasmic contents, ultimately inhibiting fungal pathogens (Di Francesco et al. 2016). Additionally, it helps determine efficacy in disease control or pest management, aiding in the selection of optimal strains for enhancing crop yield. Moreover, it guides the development of commercial biofertilizers or biopesticides, ensuring the creation of effective products that promote sustainable agriculture by reducing reliance on synthetic agrochemicals (Mena-Violante and Olalde-Portugal 2007). Overall, antagonism testing provides valuable insights into the interactions of plant probiotic bacteria with pathogens and soil microorganisms, thereby facilitating improved crop yield and quality. These proficient microbial competitors enhance plant growth either by synthesizing phytohormones or by increasing nutrient availability through secondary metabolite production (de Andrade et al. 2023).

The purpose of this study was to assess their antagonistic properties to evaluate the ability of bacteria to inhibit harmful pathogens, demonstrating their potential to protect plants from diseases. In addition, to establish beneficial effects on plant growth, plant–host interactions should be determined, and the efficacy of bacteria as plant probiotics should be evaluated via analytical techniques such as HPLC, GC‒MS, and FT-IR. This study also demonstrated the mechanisms that help in evaluating the presence of chemical compounds produced by bacteria, which may benefit plants. Overall, these tests serve to validate the potential of certain bacteria as plant probiotics, providing a scientific basis for their application in agriculture to improve plant health, resilience, and productivity.

## Materials and methods

### Isolation of fungi and purification of their DNA

The infected pod of the okra plant (*Abelmoschus esculentus*) was collected from the backyard farming source in the residential plot and exposed to surface sterilization via the addition of 1.5% sodium hypochlorite for 2 min, after which it was rinsed with water. The surface-sterilized plant tissue was placed onto the selected isolation medium, potato dextrose agar (PDA), ensuring that the infected parts were placed. PDA is composed of 1.5% agar and 2% glucose, while its nitrogen, phosphorus, vitamins, and micronutrients are sourced from a roughly filtered extract of mashed potatoes (57.5 g potatoes per liter of medium) (Gams et al. 1987). The elevated carbon-to-nutrient ratio in PDA facilitates robust growth, particularly sporulation and pigmentation across a broad spectrum of fungal species (Griffith et al. 2007). In addition, the medium was supplemented with the antibiotic streptomycin at a concentration of 100 mg/L. The isolation medium used here promoted the growth of the fungus while suppressing the growth of other microorganisms, such as bacteria. The fungal plates were incubated at 25 °C for approximately 2–5 days under suitable humid conditions. Finally, the isolated fungus was identified on the basis of morphological characteristics, such as colony appearance, color, and texture, and microscopic features, such as spore morphology. Molecular techniques such as DNA extraction were performed via a GeNei™ Fungal DNA Extraction Kit (Chuhan et al. 2022). Later, the DNA fragments were separated via agarose gel electrophoresis (AGE) (Lee et al. 2012; Drabik and Silberring 2016). The positive control used in the experiment was the 18S gene of *Candida glabrata* (Mirhendi et al. 2011; He et al. 2023). The use of a positive control helps to compare and enhance the specificity of the target organisms. For this technique, a 1 kb ladder from GCC Biotech was used. Next, PCR and 18S rRNA sequencing were employed for accurate identification via amplification of th**e** nuclear ribosomal internal transcribed spacer (ITS) region via a universal primer set: ITS 1 and ITS 4 with 5’-TCC GTA GGT GAA CCT GCG G-3’ and 5’ -TCC TCC GCT TAT TGA TAT GC-3’, respectively (White et al. 1990). Following gene amplification, the sequences were obtained via an ABI 3730XL DNA Analyzer. The acquired sequence was exported to the NCBI BLAST website. The top 10 outcome sequences of the BLAST analysis were further transferred to genome.net software, where the aligned sequences were scrutinized to detect conserved segments by providing insights into phylogenetic relationships. The alignment and phylogenetic tree were constructed via multiple alignment via fast Fourier transform (MAFFT) (Katoh and Standley 2013).

### Screening for antagonistic behavior

Antagonistic testing involves assessing the ability of one microorganism to inhibit the growth or activity of another microorganism. In the case of bacteria and fungi, antagonistic testing typically involves assessing whether certain bacteria or their products can inhibit the growth or activity of fungi (Ahemed and Kibret 2014). First, the method includes the preparation of plant probiotic bacteria (*Corynebacterium accolens* strain CNTC Th1/57*, Bacillus rugosus* strain SPB7 isolated from *Ocimum sativum, Lactobacillus pasteurii* strain DSM 23907 isolated from *Spinacia oleracea, and Cytobacillus firmus* strain NBRC 15306 isolated from *Capsicum annuum*) to perform antagonistic testing. These bacteria, which were previously identified and stored, were developed to log phase to perform antagonistic testing (Hiranmayee et al. 2023a, 2023b). The log phase was selected because it is the most active state where actively dividing cells are present and maintains the uniform physiological state of the bacteria. To obtain a log phase bacterial culture, a bacterial strain was inoculated into 10 ml of nutrient broth. This culture was incubated overnight at 37 °C in a shaker incubator (Model Labindia Instruments Labincushaker Incubator Shaker) at 150 rpm. Once the overnight culture was ready, it was diluted 1:100 in fresh medium to lower the optical density. This diluted culture was again incubated under the same conditions, and growth was monitored by measuring the optical density at 600 nm (OD600).

To assess the ability of the probiotic bacteria, the identified fungi were transferred to liquid media (PDA suspension media without agar) for further incubation. The fungal suspension was spread over the surface of nutrient agar, upon which the previously identified different plant probiotic bacteria were inoculated as a spot separately (Li et al. 2008). The plates were incubated, and the zone of inhibition around the bacterial colony was considered the result (Almoneafy et al. 2012). The bacterial isolates that presented antagonistic activity against fungi were considered for further analysis.

### Statistical analysis

To determine the bacteria with the best correlation among the four strains used, statistical application was also implemented. The statistical calculation performed here was ANOVA. ANOVA can be used to compare multiple groups simultaneously, and ANOVA not only tests for differences in means but also accounts for variability within and between groups. The results were interpreted and further analyzed on the basis of the P value as a summary of the ANOVA results.

### Extraction of secondary metabolites from plant probiotic bacteria

An inoculum of the chosen isolate was prepared in a 2000 mL conical flask containing 1000 mL of nutrient broth with optimal nutrient levels. The inoculated flasks were then incubated at 30 °C for 48 h with shaking at 200 rpm. Following incubation, the culture broth was carefully separated from the supernatant and centrifuged at 10,000 rpm for 20 min using a Sorvall X1R 100 V superspeed centrifuge designed by Thermo Fisher Scientific. Moreover, the supernatant was combined with 100% cold ethanol at a 13:1 (v/v) ratio and kept at 4 °C for 24 h to precipitate secondary metabolites. The resulting secondary metabolite pellets were recovered via centrifugation, dried at 60 °C, and purified by washing with Milli-Q water (Jayakumar et al. 2022).

### Analytical profiling of the plant probiotic bacteria

The secondary metabolites extracted earlier were subjected to a few analytical techniques, such as high-performance liquid chromatography (HPLC), gas chromatography with mass spectrometry (GC‒MS), and Fourier transform infrared spectroscopy (FT-IR). The integrated approach would provide a more detailed understanding of the bacterial metabolome. HPLC was carried out using an Agilent 1260 Infinity II instrument. A blank control where pure solvent was used without any analytes was run to check for any impurities or baseline noise. Here, the blank used was only acetonitrile. The procedure begins with sample preparation, where the sample is dissolved in an acetonitrile solvent and filtered to remove the particulates. The column used here was C18 reverse. The targeted wavelength for HPLC was 250–350 nm. The prepared sample was then injected into the HPLC system, typically through an autosampler. The sample was then transported through a chromatographic column packed with a stationary phase, where the components were separated on the basis of their interactions with the stationary phase and the mobile phase (typically a solvent or solvent mixture). The separated components are eluted from the column at different retention times and are detected by a detector, such as a UV‒Vis detector or a mass spectrometer. The resulting chromatogram provides information about the identity and quantity of the compounds present in the sample.

GC‒MS was performed via an Agilent 7890B instrument coupled with a 5977b MSD instrument. The procedure begins by running a blank where only acetonitrile was used. Later, the sample was prepared by mixing the bacterial sample with an acetonitrile solution, where the sample was extracted and filtered before injection into the GC inlet. A small volume of the prepared sample was injected into the GC injector, where it was vaporized and carried through the GC column by a carrier gas. The column used was polyethylene glycol, which measures approximately 30 m with 0.20 mm ID and a 0.2 µm thin film, to reduce peak broadening and enhance resolution. As the temperature increased, the compounds were separated on the basis of their volatility and physio-chemical properties. As the separated compounds exit the GC column, they enter the mass spectrometer where they are ionized by electron impact (EI) or chemical ionization (CI). The ions were then accelerated and separated on the basis of their mass‒charge ratio (m/z) before being detected. The resulting mass spectra provided information about the identity and quantity of the compounds present in the sample.

FT-IR is a technique used to analyze the chemical composition of materials on the basis of their interaction with infrared light. In this process, the bacterial pellet is typically mixed with a solvent that does not interfere with the IR spectra and effectively disperses the sample for analysis. Therefore, a small amount of sample was dissolved in a potassium bromide solvent. It was then placed on sodium chloride disks, which acted as suitable substrates. The sample was placed in the designated compartment, and FT-IR analysis was performed. Scanning was performed over a range of wavenumbers (typically 4000–400 cm⁻^1^) to collect the infrared spectra, which indicate the sample's absorbance or transmittance. The FT-IR instrument emits infrared radiation through the sample, and the detector measures the intensity of transmitted or reflected light at different wavelengths. Once the data acquisition was completed, the resulting spectrum was analyzed via appropriate software provided by the instrument manufacturer software. The characteristic peaks in the spectrum corresponding to functional groups or molecular vibrations present in the sample were identified. A comparison of the resulting peaks with reference spectra was performed. Finally, the sample compartment and other accessories were used to prevent cross-contamination for future analyses.

## Results

### Morphological and microscopic identification of the fungi

**Fig. 1:**
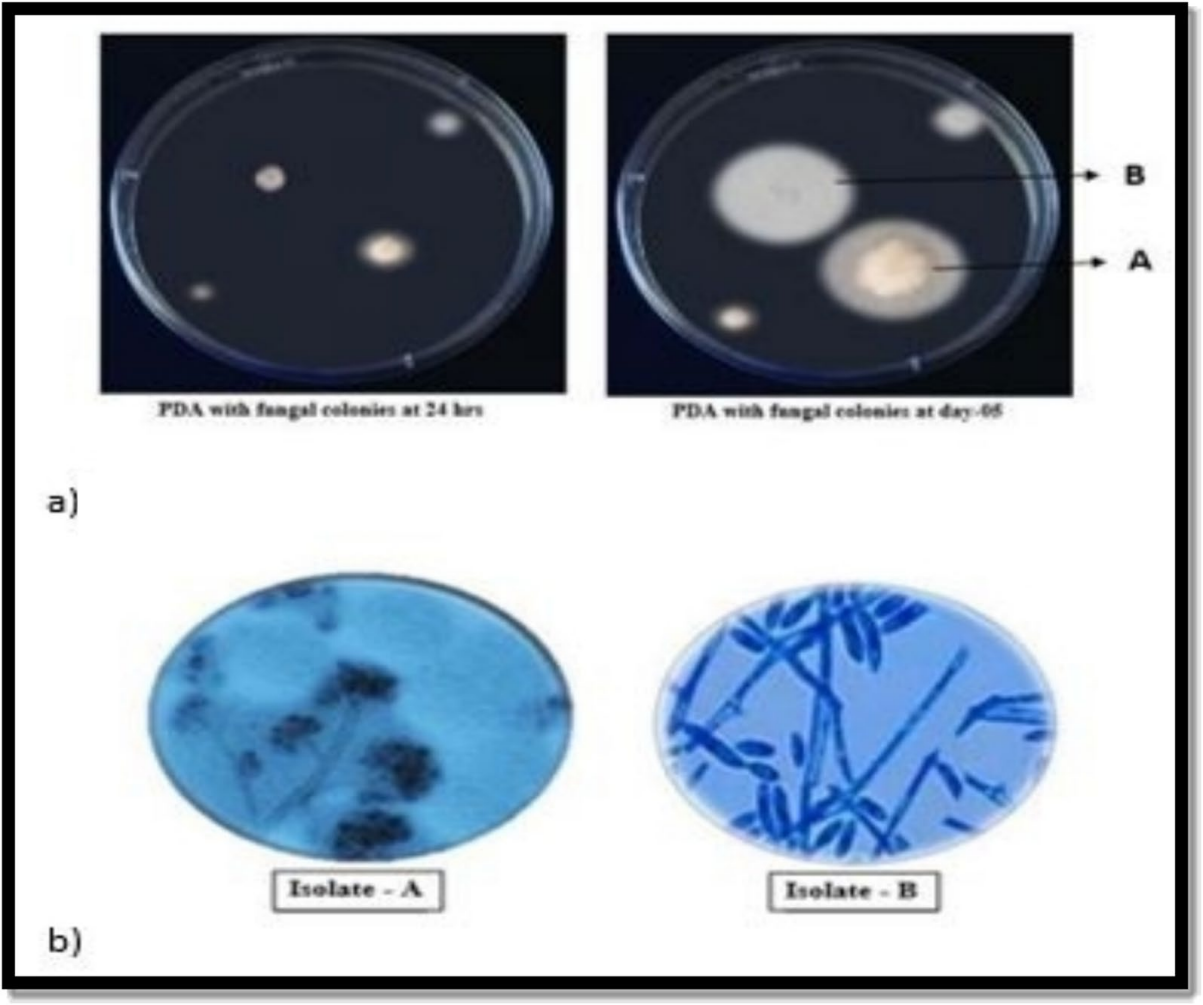
**a** Isolation of fungi from the infected pods of *Abelmoschus esculentus* via PDA medium, which was incubated for 5 days. **b** Microscopic observation of isolated fungi at 45X magnification

Initially, 2 different colonies were observed and labeled A and B, as shown in Fig. 1a). Isolate A appeared to be fluffy and cottony in texture at the center. As the colonies matured, they became denser. The color of the colonies ranged from white to gray. The surface of the colonies may become covered with a fine, powdery layer of conidia as the fungus matures. When viewed from the underside, colonies often exhibit dark gray to black coloration due to the formation of sclerotia (compact masses of mycelium). Isolate-B grew faster than Isolate-A did. This colony appeared to be fluffy or cottony in texture. Beneath the surface of the colony, a dense network of hyphae forms the underlying mycelium.

The fungal isolates typically exhibited a relatively rapid growth rate on PDA media. The colonies were stained with lactophenol cotton blue and further examined under a microscope at 45X magnification, as shown in Fig. 1b). Isolate A presented hyphae that were typically branched, forming a network of mycelia. The conidiophores were erect, unbranched or sparsely branched, and raised directly from the mycelium. They were usually hyaline (colorless) and cylindrical in shape. Isolate-B had branched, septate conidiophores that were singly or in clusters from the mycelium. The conidia were hyaline (transparent), single-celled, and varied in shape; they were typically fusiform or cylindrical with rounded ends and were slightly curved.0

### Molecular analysis

**Fig. 2:**
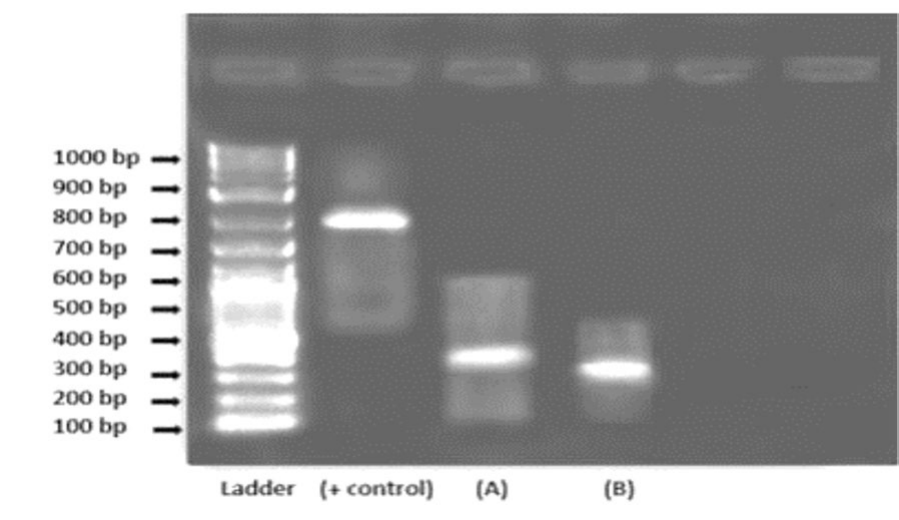
Gel amplicon picture showing the results corresponding to (**A**) *Botrytis cinerea* and (**B**) *Fusarium oxysporum* when compared with a positive control i.e., the 18S gene of *Candida glabrata* and use of 1 Kb DNA ladder

**Fig. 3:**
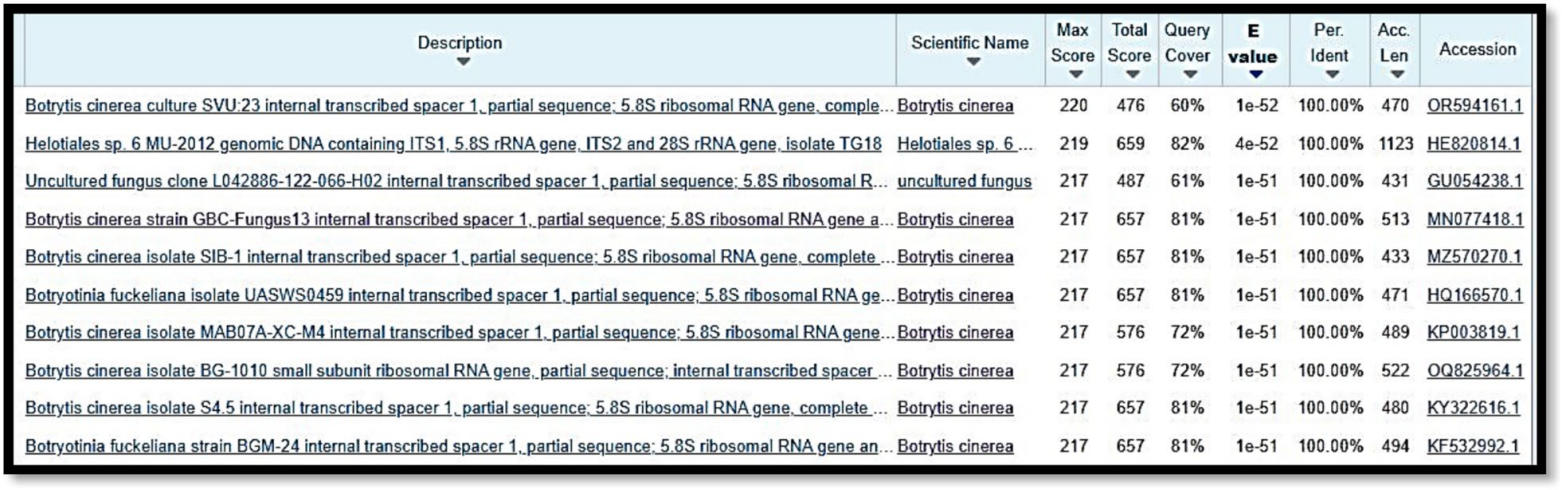
BLASTn of Sample Isolate-A (*Botrytis cinerea*)

**Fig. 4:**
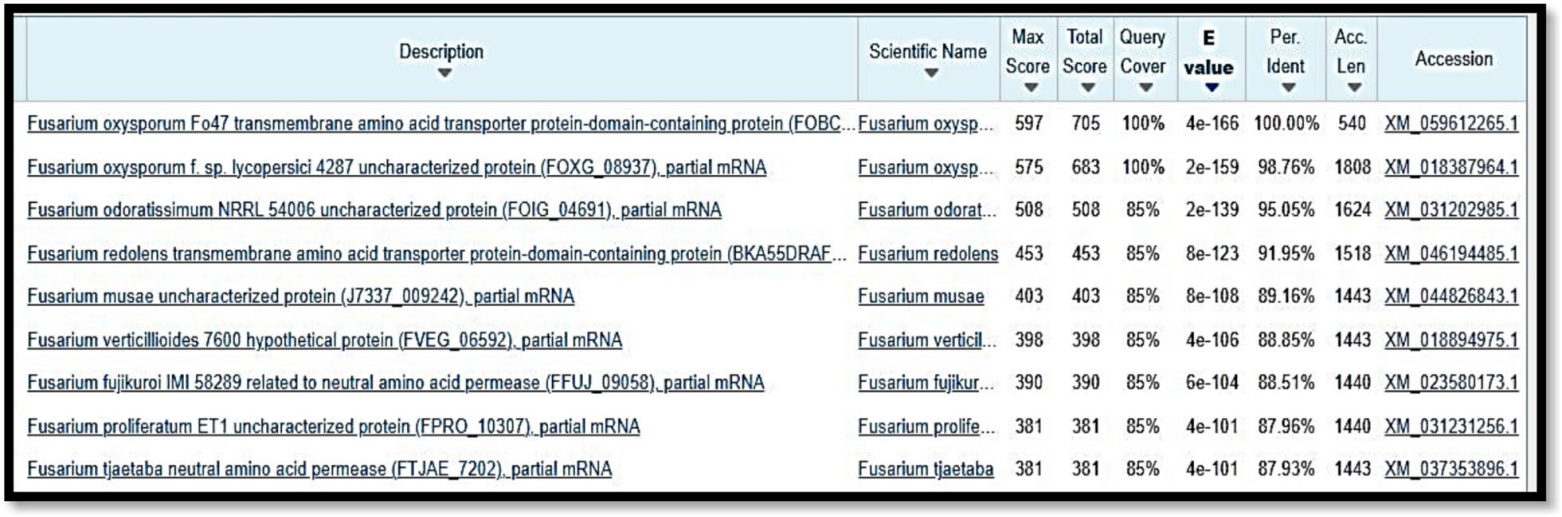
BLASTn of Sample Isolate-B (*Fusarium oxysporum*)

The gel amplicon images of the isolates revealed bands at the 300-bp and 400-bp ranges approximately are shown in Fig. 2. On the basis of the BLAST results as shown in Fig. 3 and Fig. 4, isolate A and isolate B were found to be *Botrytis cinerea* and *Fusarium oxysporum,* with OR594161.1 and XM_059612255.1 accession numbers, respectively.

#### Sequences obtained for isolate-A

ATCGGTGCGGTTCTGCGGGCCACCCTTGTGTATTATTACTTTGTTGCTTTGGCGAGCTGCCTTCGGGCCTATTGAGTCTATGTCAGTAATGGCAGGCTCTAAAATCAGTGGCGGTTTGTTGCTTTGGCGAGCTGCCTTCGGGCCTTGTATGCTCCGATAAGTAATGTGAATTGCAGAATTCAGTGAATCATCGAATCTTTGAACGCACATTGCGCCCCTTGGTATTCCGGGGGGCATGCCTGTTCGAGCGTCATTTCAACCCTCAAGCTTAATCATCGAATCTTCGTTACAGGTTCTCGGTGTGCTTCCGTTACAGGTTCTCGGTGTGCTTCCGAAATGCGATAAGTAATGTGAATTGCAGAATTCAGTGAATCATCGAATCTTTGAACGCACATTGCGCCCCTTGGTATTCCGGGGGGCATGCCTGTTCGA.

#### Sequence obtained for Isolate-B

TTGTGTTGAGGGCAATTGACAGGGTGACCATAGCTGAAGCCGCGACGAAGATGACTTGTATCATCATCATGACACCTGTGATGCTCTCCAGGGTTCTTCCACCGACGAAGCGGGTCATGTCGACGATGTTCAATACCTGAGGGTGCAGCCTGTAGTATTGTAACAGCTCATACGCTGTATACCATGATATCACACCGATGCCGATAAGCGCGATAATACCGGGAACGAGGCCGAGGGTCTTGAGGGTCGAGGGAAGAGACAAGATACCAAGACCGATCTGATTGGCAAATAGGATAAATATGGTATCCCATCGCTTCAGTGTTTTCAATACCTGAGGGTGCAGCCTGTAGTATTGTAACAGCTCATACGCTGTATACCATG.

**Fig. 5:**
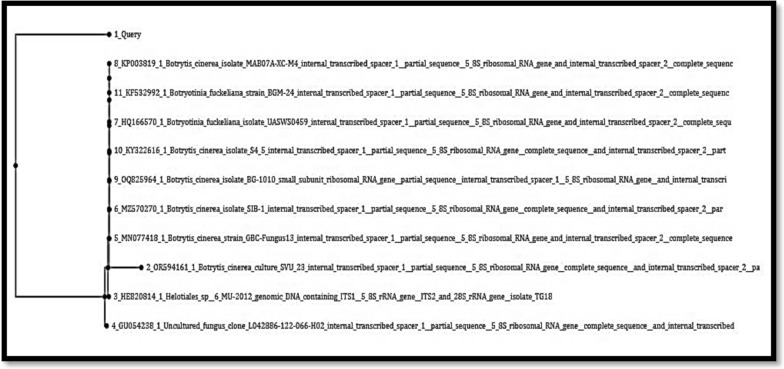
Phylogenetic tree for Sample Isolate-A

**Fig. 6:**
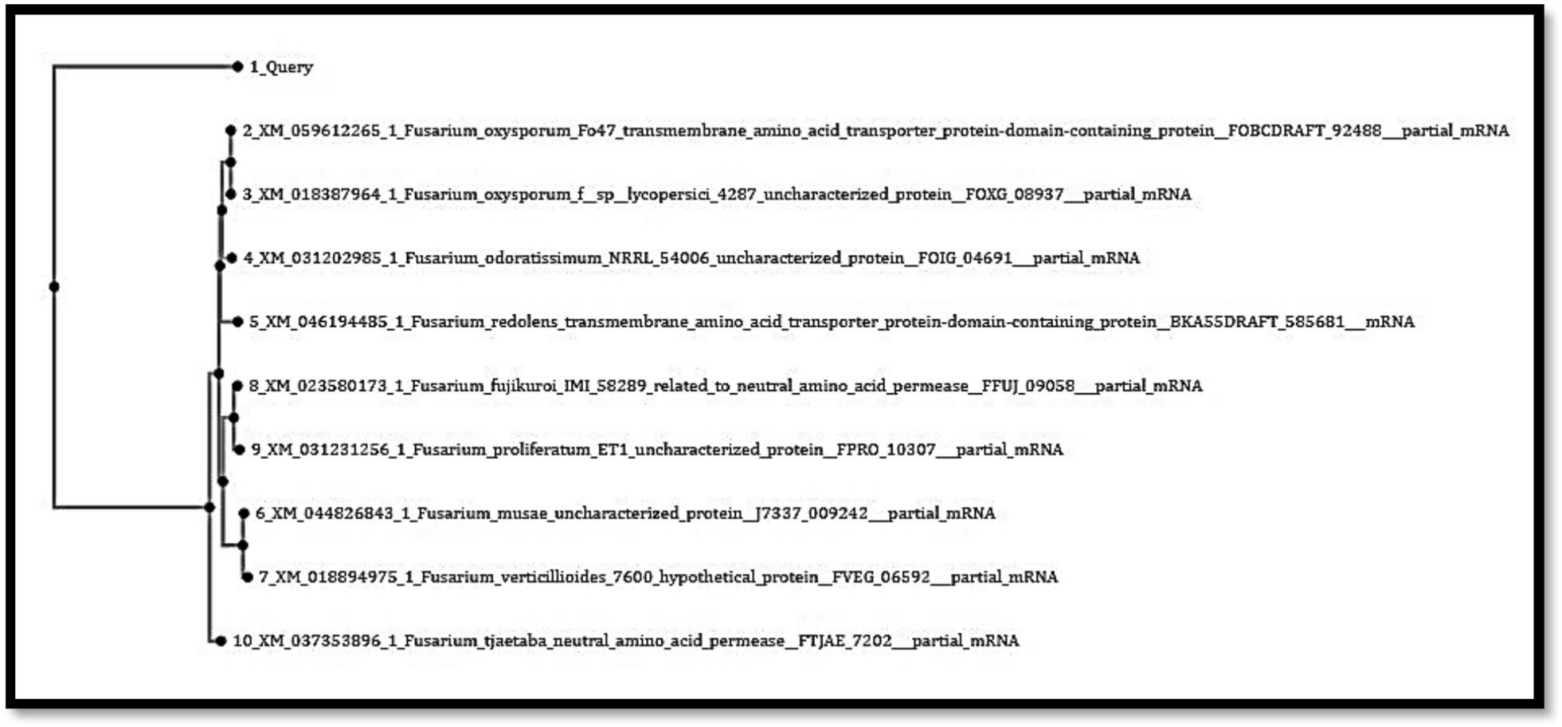
Phylogenetic tree for Sample Isolate-B

The query in the phylogenetic tree shows its branch connecting to the closely associated fungal strain as shown in Figs. 5 and 6.

Here the query sequence is more closely associated to OR594161.1 indicating the relatedness between the organisms. The clustering suggests that the *Botrytis cinerea* isolates in the tree are genetically similar, confirming their placement in the same clade.

Here the query sequence is more closely related to XM_059612255.1 indicating the common features. The branching pattern indicates that your query sequence belongs to the *Fusarium oxysporum* complex, which includes important plant pathogens.

### Antagonistic behaviour

**Fig. 7:**
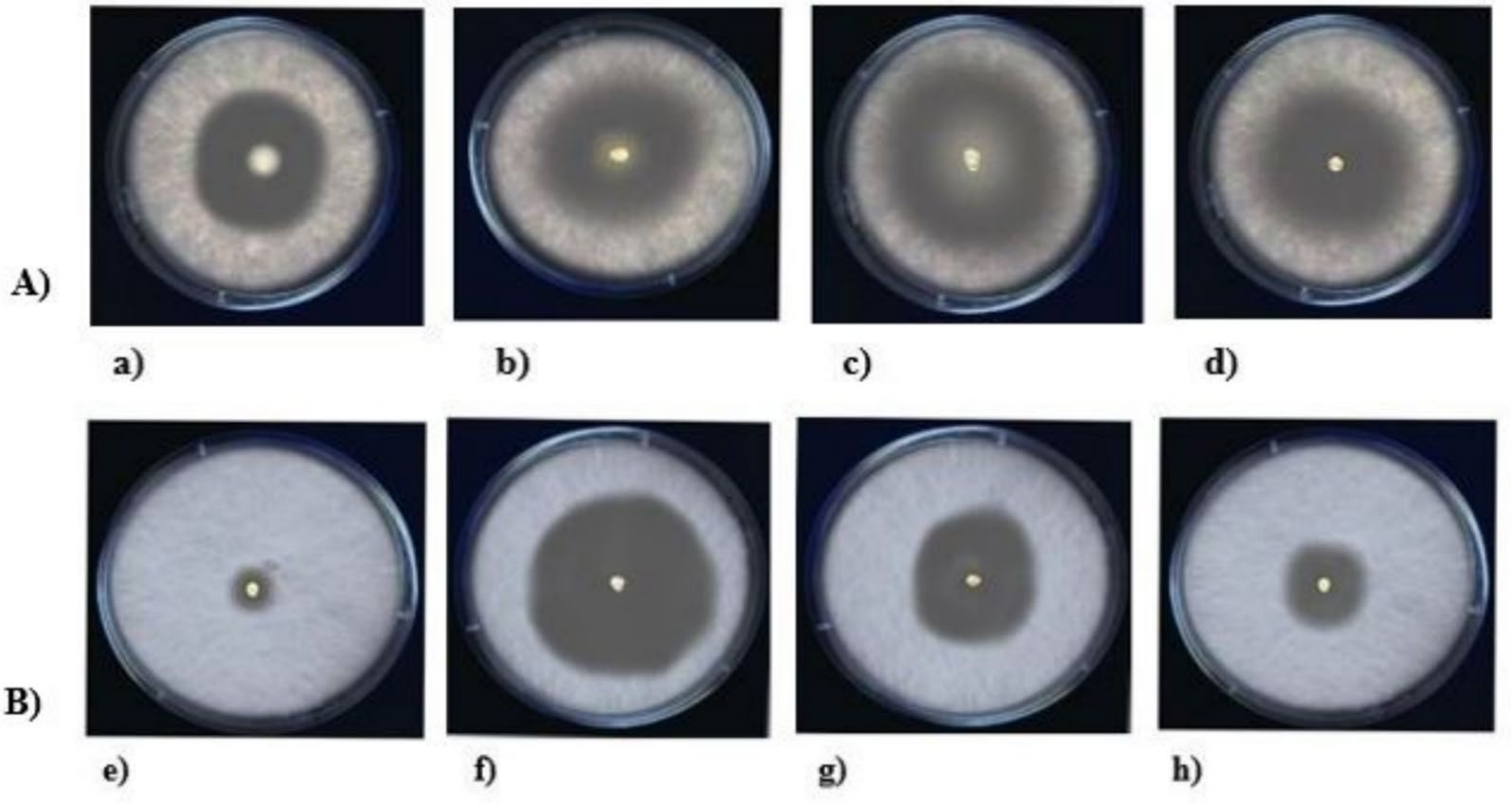
Antagonistic activity of plant probiotic bacteria against *Botrytis cinerea* and *Fusarium oxysporum*. **A** Zone of inhibition shown by plant probiotic bacteria, namely, a) *Corynebacterium accolens* strain CNTC Th1/57, b) *Bacillus rugosus* strain SPB7, c) *Lactobacillus pasteurii* DSM 23907, and d) *Cytobacillus firmus* strain NBRC 15306, against *Botrytis cinerea* fungal lawn. **B** Zone of inhibition shown by plant probiotic bacteria, namely, e) *Corynebacterium accolens* strain CNTC Th1/57, f) *Bacillus rugosus* strain SPB7, g) *Lactobacillus pasteurii* DSM 23907, and h) *Cytobacillus firmus* strain NBRC 15306, against *F. oxysporum* fungal lawn

The antagonistic effects of fungal organisms, i.e., *Botrytis cinerea* and *Fusarium oxysporum,* were evaluated with previously identified plant probiotic bacteria. The bacteria that inhibited the growth of the fungus and formed a zone of inhibition were considered the strains with the best antagonistic properties. An organism with a smaller zone was ruled out, and the remaining organisms were removed for analytical assessment. As shown in Fig. 7, the bacterial strains *Bacillus rugosus* strain SPB7 and *Lactobacillus pasteurii* DSM 23907 formed a relatively large zone, which was confirmed by their ability to inhibit fungal activity. The zones of inhibition were measured as 11 mm and 25 mm for *Botrytis cinerea* and 19 mm and 12 mm for *Fusarium oxysporum*, as tabulated in Table 1.

**Table 1:** Zone of inhibition of bacteria against fungi

Bacterial Strain	Zone of inhibition against *Botrytis cinerea*(mm)	Zone of inhibition against *Fusarium oxysporum (*mm)
*Corynebacterium accolens* strain CNTC Th1/57	9	2
*Bacillus rugosus* strain SPB7	11	19
*Lactobacillus pasteurii* DSM 23907	25	12
*Cytobacillus firmus* strain NBRC 15306	9	5

### Statistical validation

The results obtained from ANOVA revealed an F statistic of 12.212, with a p value of 0.0129, indicating a statistically significant difference in the zones of inhibition among the different bacterial strains against *Botrytis cinerea.* Since the p value was less than the significance level of 0.05, we rejected the null hypothesis. This finding indicates that at least one bacterial strain had a significantly different zone of inhibition than the other strains did.

The F statistic of 6.26, with a p value of 0.04640, indicates an even stronger statistically significant difference among the bacterial strains against *F. oxysporum.* The very low p value reinforces that there were significant differences, leading to the rejection of the null hypothesis. These findings indicate that some bacterial strains are notably more effective at inhibiting the growth of *F. oxysporum* than others are. The results suggest that both *Botrytis cinerea. Fusarium oxysporum* was significantly affected by the bacterial strains tested, indicating the potential for the use of these strains as biocontrol agents in agricultural settings.

### Analytical techniques

On the basis of the statistical results, the bacterial strains *Bacillus rugosus* strain SPB7 and *Lactobacillus pasteurii* DSM 23907 were further subjected to analytical techniques to detect the presence of various compounds. HPLC for *Bacillus rugosus* strain SPB7 and *Lactobacillus pasteurii* DSM 23907 resulted in chromatograms where the smaller molecules penetrated into the pores of the stationary phase more easily and were thus retained for shorter periods, resulting in earlier elution times. Conversely, larger molecules experience more hindered penetration into the stationary phase and are retained for longer, eluting later in the chromatogram.

**Fig. 8:**
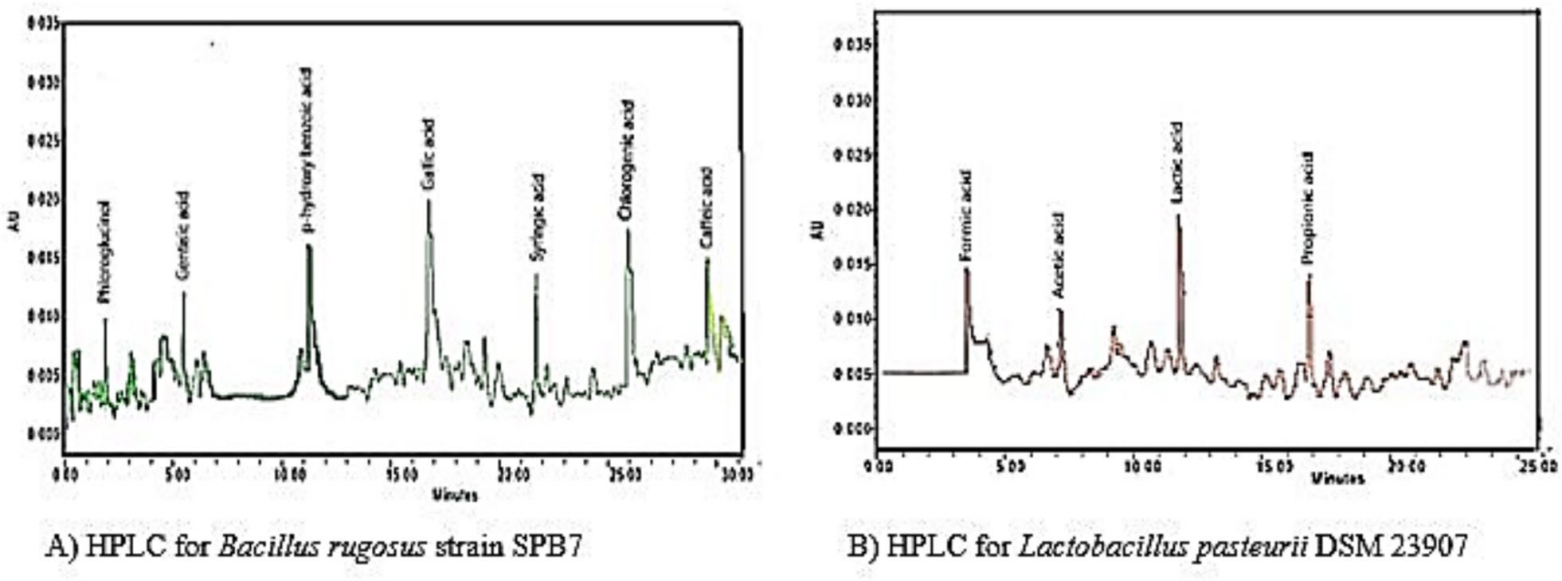
HPLC

As shown in Fig. 8, the *Bacillus rugosus* strain SPB7 presented peaks indicating the presence of different phenolic compounds, such as phloroglucinol, gentisic acid, para-hydroxybenzoic acid, gallic acid, syringic acid, chlorogenic acid and caffeic acid, with specific retention times, and *Lactobacillus pasteurii DSM 23907* presented peaks corresponding to the presence of different organic acids within the strain at a specific retention time.

**Fig. 9:**
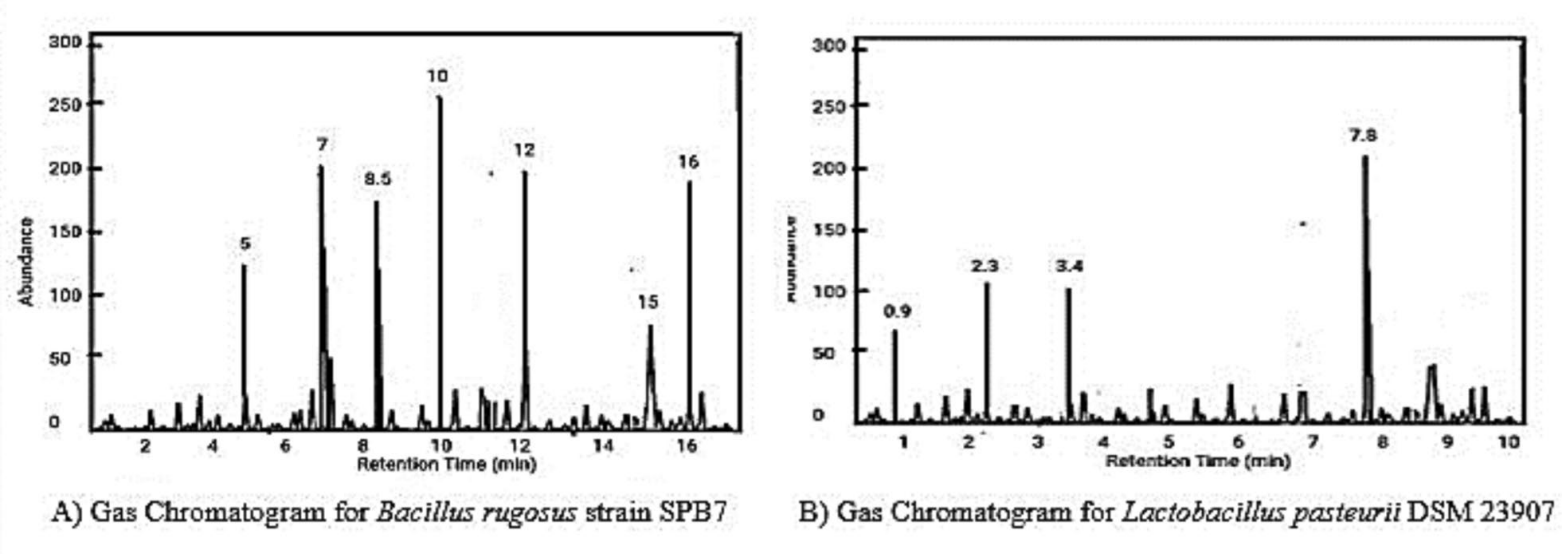
Gas Chromatogram from the GC‒MS Analysis

To detect the trace elements of the compounds present in the bacterial isolates, GC‒MS was performed. Here, the gas chromatogram graph shows the separation of different compounds on the basis of their retention times. Each peak represents a different compound, and the area under the peak correlates with the quantity of that compound present in the sample, as shown in Fig. 9. At approximately 5 min, 7 min, 8.5 min, 10 min, 12 min, 15 min and 16 min, the compounds phloroglucinol, gentisic acid, p-hydroxybenzoic acid, gallic acid, syringic acid, chlorogenic acid and caffeic acid were detected with respect to *Bacillus rugosus* strain SPB7. With respect to *Lactobacillus pasteurii* DSM 23907, formic acid, acetic acid, propionic acid and lactic acid were detected at 0.9 min, 2.3 min, and 3. 4 min and 7.8 min, respectively.

**Fig. 10:**
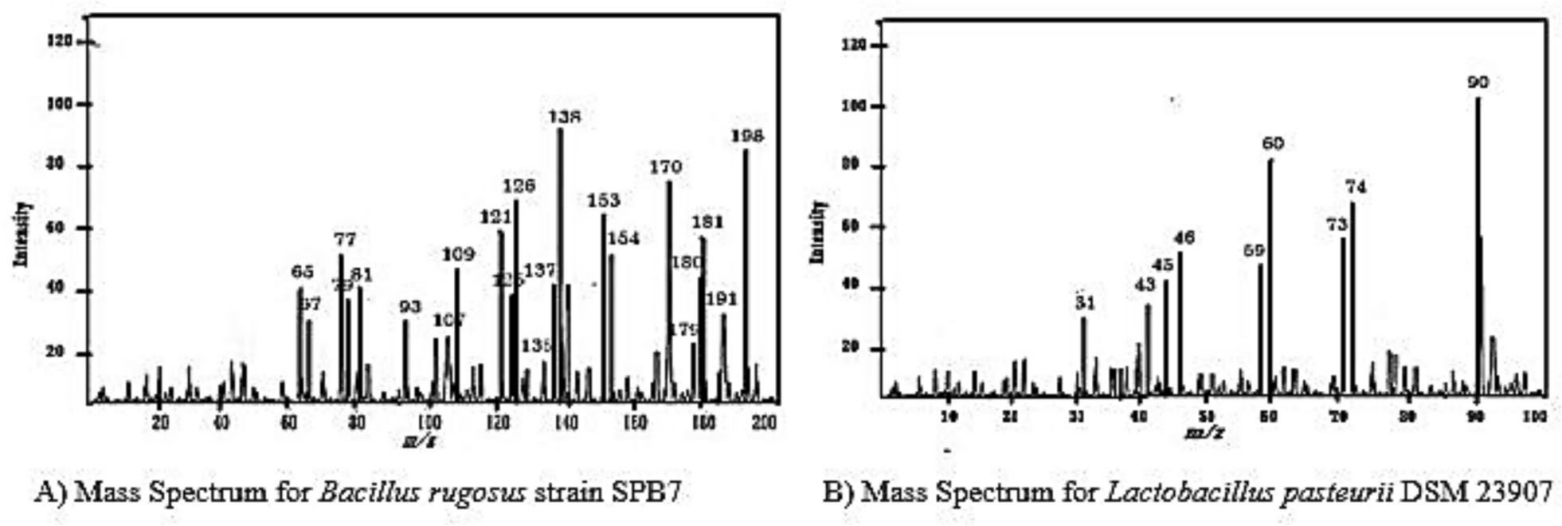
Mass spectrum within GC–MS Analysis

The mass spectrum graph provides information about the mass‒charge ratio (m/z) of ions detected by the mass spectrometer as shown in the Fig. 10. This study provides insight into the molecular structure of these compounds. The peaks in the mass spectrum correspond to the molecular fragments of the compounds present in the sample.

The final results of GC‒MS corresponding to *Bacillus rugosus* strain SPB7 confirmed the presence of phenolic compounds, and *Lactobacillus pasteurii* DSM 23907 confirmed the presence of organic acids with different concentrations and retention times through chromatography. These compounds were even confirmed by their fragment ions through mass spectra, as shown in Tables 2 and 3. The peaks formed at different m/z ratios were compared with the standard database to identify the structural similarities of the compounds. Finally, all the compounds were identified via MassHunter, which confirmed the presence of the compounds.

**Table 2:** Compound identification results of *Bacillus rugosus* strain SPB7 corresponding to the GC‒MS mass spectrum

Compound Name	Molecular peak(m/z ratio)
Phloroglucinol	12
2,4,6-Trihydroxybenzene	109
2,4,6-Trihydroxytoluene	81
2,4-Dihydroxytoluene	79
Hydroxybenzyl radical	67
Gentisic acid	154
2,5 dihydroxy benzoic acid	137
2-Hydroxybenzoic acid (salicylic acid)	109
2-Carboxybenzaldehyde	93
Hydroxybenzaldehyde	65
p-hydroxy benzoic acid	138
4-hydroxybenzoic acid	121
Benzoic acid	93
Phenol	77
Gallic acid	170
3,4,5-trihydroxybenzoic acid	153
3,4-Dihydroxybenzoic acid	125
3-Hydroxybenzoic acid (protocatechuic acid)	109
Hydroxybenzoic acid	81
Syringic acid	198
4-Hydroxy-3,5-dimethoxybenzoate ion	181
3-Methoxy-4-hydroxybenzoate ion	109
3,5-Dimethoxybenzoate ion	137
4-Hydroxy-3,5-dimethoxybenzoate ion	153
Caffeic acid	191
Quinic acid	179
Caffeoyl fragment	135
Caffeic acid	180
Caffeic acid	135
4-Hydroxycinnamic acid	107
Cinnamic acid	93

**Table 3:** Compound identification results of *Lactobacillus pasteurii* DSM 23907 corresponding to the GC‒MS mass spectrum

Compound Name	Molecular peak(m/z ratio)
Formic acid radical cation	46
Formic acid (methanoic acid)	45
Acetic acid (ethanoic acid)	60
Acetyl radical cation	45
Acetyl fragment ion	43
Methyl cation	31
2-hydroxypropanoic acid(Lactic acid0	90
Acetaldehyde (ethanal)	73
Acetone	60
Propionic acid (propanoic acid)	74
Propionyl cation	59
Propionyl fragment	45
Propyl cation	31

**Fig.11:**
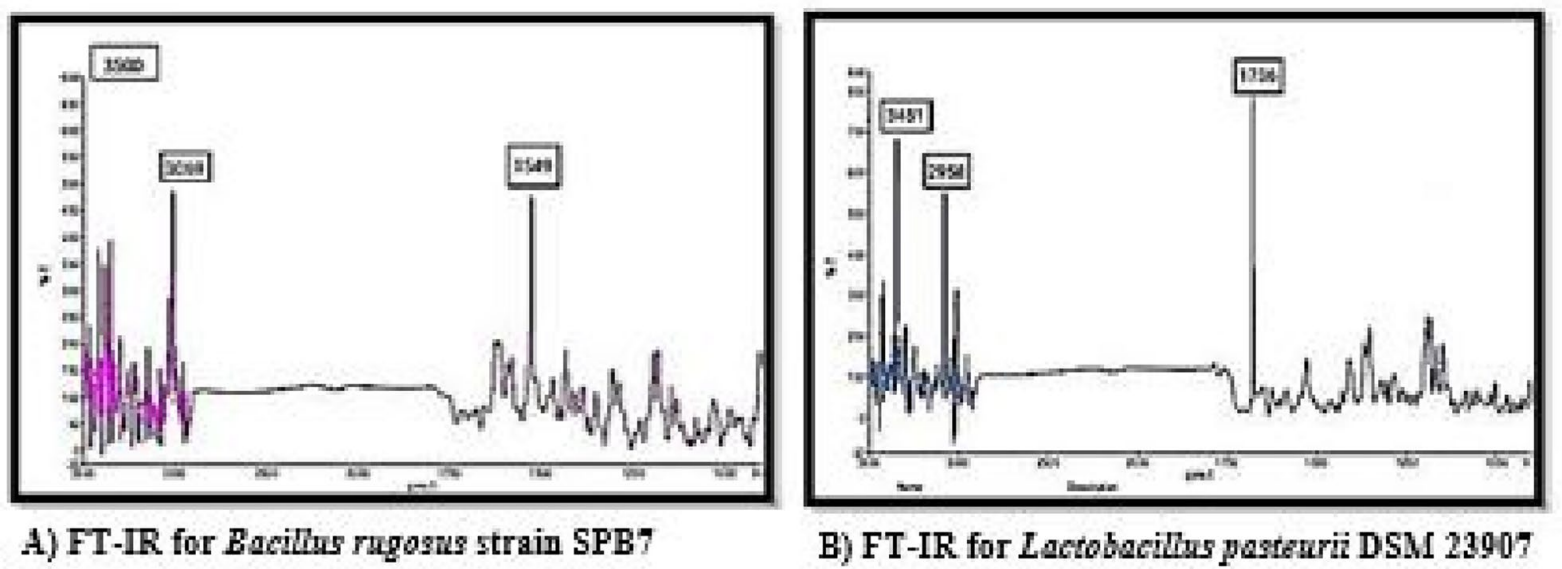
FT-IR

Next, the functional groups of the isolates were evaluated via FT-IR. The peak at 3500 cm^−1^ associated with the *Bacillus rugosus* strain SPB7 clearly indicates the presence of hydroxyl functional groups, resulting in compounds such as alcohols and phenols. To confirm the presence of phenolic compounds, in addition to O–H, aromatic C-H and aromatic C = C stretching vibrations were observed at 3069 cm^−1^ and 1549 cm^−1^, respectively, as shown in Fig. 11.

When exposed to FT-IR, Lactobacillus pasteurii DSM 23907 clearly presented a peak at 1736 cm^−1^, which corresponds to the carboxyl group (-COOH) as the functional group. Further confirmation was performed by observing the other absorption bands at 3451 cm^−1^ and 2958 cm^−1^, indicating hydroxyl group (O–H) and alkyl group (C-H) stretching vibrations, respectively.

Thus, considering the results of the analytical techniques, the bacteria *Bacillus rugosus* strain SPB7 and *Lactobacillus pasteurii* DSM 23907 were able to produce phenols and organic acids, respectively.

## Discussion

This study investigated the effectiveness of plant probiotic bacteria against common plant pathogenic fungi, such as *Botrytis cinerea* and *Fusarium oxysporum*, and demonstrated their ability to inhibit fungal growth. Later on its approach towards the analytical techniques for the compound identification within the plant probiotic bacteria. This integrated methodology of antagonistic testing and analytical methods using high-resolution data with accurate compound identification was a novel approach. According to the present study, *Bacillus rugosus* strain SPB7 and *Lactobacillus pasteurii* DSM 23907 exhibited stronger antagonistic behavior against pathogenic fungi than the other bacteria examined. To understand the basis of this behavior, analytical methods such as HPLC and GC coupled with MS were employed to identify and confirm compounds within plant probiotic bacteria (PPB) samples. This integrated methodology of antagonistic testing and analytical methods using high-resolution data with accurate compound identification was a novel approach. The HPLC excels at separating and quantifying biomolecules, including metabolites and antibiotics, thereby offering valuable insights into a sample’s biochemical profile. GC‒MS was used because it is effective for examining volatile compounds such as fatty acids and secondary metabolites, enabling the identification of specific metabolic products. FT-IR complements these techniques by providing details about the molecular structure and functional groups in bacterial cells, aiding in the characterization of bacterial species. Among the analytical methods, mass spectrometry (MS) methods have gained popularity for microbial typing because of their speed, cost-effectiveness, simplicity, and applicability across various microorganisms, including bacteria, Archaea, and fungi (Calvigioni et al. 2023). By integrating these methods, we obtained a more comprehensive perspective of the bacterial sample, improving accuracy, validating findings, and revealing few metabolic interactions. Our findings revealed the compounds that play a significant role in plant-related properties. For example, phenols can scavenge reactive oxygen species, thereby protecting plants from oxidative stress, and can also modify the rhizosphere microbiome, promoting the growth of beneficial microbes (Aboul-Enein et al. 2007; Saini.^2024^). Organic acids can solubilize minerals, making them available to plants and helping reduce disease susceptibility in plants by altering the soil pH (Lopez-Bucio et al. 2000; Panchal et al. 2021).

Plant probiotic bacteria (PPB) show promise in reducing agricultural chemical usage (e.g., fertilizers, pesticides), potentially enhancing quality while lowering costs and promoting sustainable agriculture (Jimenez-Gomez et al. 2018). Overall, the multifaceted role of identifying plant probiotic bacteria involves microbiological, biochemical, molecular, and ecological approaches to harness beneficial interactions for sustainable agriculture. In addition to the above findings, the other important aspect of plant probiotic bacteria that could be used to screen further is oil degradation by the bacteria. Biosurfactant-producing bacteria are utilized significantly in microbial enhanced oil recovery (MEOR), specifically in the biosparging process. In addition to MEOR, these bacteria play additional roles, such as eliminating pathogens within plants and increasing the availability of plant-associated bacteria for improved plant health (Golamari 2023).

Several studies have also underscored the significant role played by plant probiotics. As a result, increasing microbial diversity has the potential to increase soil fertility and restore the acquisition of various beneficial services at the community level, which are crucial for strong plant development (Romero et al. 2023). This study was motivated by prior agronomic research highlighting plant probiotics as promising means to improve soil quality. Its application in agriculture improves nutrient availability and enhances resistance to biotic stress. Finally, the resulting strains could be further evaluated in pilot trials aimed at developing a new formulation to protect plants from pathogen attack. Techniques such as biopriming, which involves treating seeds with these beneficial microorganisms to promote their growth and establishment in the plant's environment, may be considered in this evaluation. This research ultimately underscores the potential of beneficial microbiomes to interact with host plants through chemical-triggered signaling pathways, supporting the prioritization of organic agriculture for improved sustainability in food production and crop protection.

## Conclusion

Research has demonstrated that certain bacteria produce phenols and organic acids, which offer several benefits to plants. These compounds improve soil health by facilitating organic matter decomposition, increasing nutrient availability, and promoting plant growth through improved root development and nutrient uptake. Phenols also possess antimicrobial properties that help protect plants from pathogens, while some inhibit the growth of weeds and competing plants. Additionally, these bacteria form symbiotic relationships, such as nitrogen fixation, which enhances nutrient exchange, and produce secondary metabolites that act as signaling molecules to bolster plant defenses. These interactions contribute to healthier ecosystems, supporting plant growth and resilience. Phenols act as antioxidants, modulating plant hormone levels to improve stress tolerance, whereas organic acids alter the pH of the rhizosphere, affecting nutrient availability and microbial communities. The allelochemicals produced by bacteria may also suppress weed growth, suggesting a natural weed management strategy. The choice of bacteria depends on factors such as target plant species, soil conditions, and desired outcomes, such as disease resistance or enhanced nutrient uptake. The successful integration of these bacteria into agricultural practices requires careful formulation to account for their effects on plant physiology and soil microbial communities, potentially leading to sustainable agricultural practices that increase crop productivity while minimizing environmental impacts.

## Data Availability

The published article includes all data generated or analyzed during this study.

## Abbreviations

AGE: Agarose Gel Electrophoresis
ANOVA: Analysis of Variance
BLAST: Basic Local Alignment Search Tool
DNA: Deoxyribose Nucleic acid
FT-IR: Fourier Transform Infrared Spectroscopy
GC–MS: Gas Chromatography coupled with Mass Spectrometry
HPLC: High Performance Liquid Chromatography
ITS: Internal Transcribed Spacer
MAFFT: Multiple Alignment using Fast Fourier Transform
MEOR: Microbial Enhanced Oil Recovery
MS: Mass Spectroscopy
MSD: Mass Selective Detector
NCBI: National Centre for Biotechnology Information
PDA: Potato Dextrose Agar
PGPR: Plant Growth Promoting Rhizobacteria
PPB: Plant Probiotic Bacteria
UV–Vis Detector: Ultraviolet and visible light detector
